# Prevalence, risk factors, and complications associated with hyponatraemia following elective primary hip and knee arthroplasty

**DOI:** 10.1186/s13741-021-00197-1

**Published:** 2021-08-03

**Authors:** Emma Cunningham, Nicola Gallagher, Paul Hamilton, Leeann Bryce, David Beverland

**Affiliations:** 1 Centre for Public Health (Queen’s University, Belfast), Institute of Clinical Sciences, Royal Victoria Hospital, Belfast, BT12 6BA Northern Ireland; 2 Outcomes Unit, Musgrave Park Hospital, Belfast Health and Social Care Trust, Stockman’s Lane, Belfast, BT9 7JB Northern Ireland; 3 Centre for Medical Education, Queen’s University Belfast, Mulhouse Road, Belfast, BT12 6BA Northern Ireland; 4 Department of Clinical Biochemistry, Royal Victoria Hospital, Belfast, BT12 6BA Northern Ireland

**Keywords:** Hyponatraemia, Sodium, Prevalence, Risk factors, Total hip arthroplasty, Total knee arthroplasty, Uni-compartmental knee arthroplasty

## Abstract

**Background:**

Hyponatraemia, defined as a serum sodium [Na] concentration below 135 mmol/L, is common following surgery. As inpatient peri-operative stays shorten, there is a need to recognise pre-operative risk factors for post-operative hyponatraemia and complications associated with a peri-operative drop in Na. This audit aimed to investigate the prevalence of, risk factors for, and complications associated with hyponatraemia following elective primary hip and knee arthroplasty.

**Methods:**

Data were collected within a retrospective audit of inpatient complications and unplanned reattendance or readmission at hospital in consecutive elective primary hip and knee arthroplasty patients in a single high throughput elective primary joint unit. The hospital’s electronic database identified 1000 patients who were admitted electively between February 2012 and June 2013 under the care of a single consultant orthopaedic surgeon for either total hip arthroplasty, total knee arthroplasty, or uni-compartmental knee arthroplasty. Groups were compared using appropriate tests, including chi-square analysis (or Fisher’s exact test), Mann-Whitney U test, Kruskal-Wallis test, and Wilcoxin signed-rank test. Logistic regression analysis was used to determine factors associated with hyponatraemia.

**Results:**

Of the total 1000 patients, 217 (21.7%) developed post-operative hyponatraemia. Of these, 177 (81.6%) had mild (Na 130–134 mmol/L), 37 (17.1%) had moderate (Na 125–129 mmol/L), and 3 (1.4%) had severe (Na < 125 mmol/L) hyponatraemia. In multivariate analysis, age, pre-operative Na, and fasting glucose on day 1 remained significantly associated with having hyponatraemia post-operatively. There were no significant differences in reattendance at emergency departments and/or readmission within 90 days between those who had post-operative hyponatraemia whilst in hospital (39/217 = 18.0%) and those who did not (103/783 = 13.2%), or between those who were discharged with hyponatraemia (18/108 = 16.7%) and those discharged with normal Na (124/880 = 14.1%).

**Conclusion:**

Approximately one fifth of elective joint arthroplasty patients had post-operative hyponatraemia. In these patients, older age, lower pre-operative Na and higher fasting glucose predicted post-operative hyponatraemia. We found no evidence that those discharged with hyponatraemia had more reattendance at emergency departments or readmission to hospital. We suggest that otherwise well patients with mild hyponatraemia can safely be discharged and followed up in the community.

**Supplementary Information:**

The online version contains supplementary material available at 10.1186/s13741-021-00197-1.

## Introduction

Hyponatraemia, defined here as a serum sodium [Na] concentration below 135 mmol/L, is common following surgery (Spasovski et al., 2014; Rudge et al., 2014). In vivo regulation of Na and water is complex, and surgery may lead to a reduced Na concentration via several pathways. These likely include blood loss leading to stimulation of baroreceptors and appropriate production of antidiuretic hormone (ADH), the inflammation cascade (Park and Shin, 2013), pain, vomiting, fever, positive pressure ventilation, anaesthesia, and analgesia (Hennrikus et al., 2015; Upadhyay et al., 2006; Biswas and Davies, 2007). Many of the latter are linked with so-called ‘inappropriate’ ADH release, accounting for around one third of hyponatraemia cases. Hyponatraemia is caused by an excess of total body water when compared to total body sodium content; therefore, volume status and osmolality play a key role (Rondon and Badireddy, 2021).

Whilst a small, transient, post-operative drop in Na may be normal and appropriate (estimates range from 0.2 to 3 mmol, with duration less than 48 h) (Hennrikus et al., 2015; Harris et al., 2015), a significant hyponatraemia may be accompanied with cerebral oedema and associated morbidity and mortality. Hyponatraemia may also indicate underlying physiological vulnerabilities, being associated as it is, with increasing age, comorbidity, and polypharmacy (Udy et al., 2005; Hawkins et al., 2003; Mohan et al., 2013; Bissram et al., 2007). Similarly, it is not clear whether the associations with increased length of stay (LOS) and mortality after surgery (Hennrikus et al., 2015; Nagler et al., 2014; Madsen et al., 2016; Tinning et al., 2015) are due to the effects of hyponatraemia or to associated issues. For example, Leung et al. (2012) have shown that low pre-operative Na is associated with negative outcomes after surgery, suggesting that both the hyponatraemia itself and the negative outcomes may reflect underlying problems (Leung et al., 2012).

Existing studies of post-operative hyponatraemia have tended to encompass different surgical groups, for example, combining elective and emergency populations (Hennrikus et al., 2015; Harris et al., 2015; McCausland et al., 2014; Cecconi et al., 2016), with very few concentrating on elective arthroplasty populations (Abola et al., 2019; Sah, 2014; Guglielminotti et al., 2003).

Current surgical practices are less likely to induce hyponatraemia (e.g. surgeries are shorter and blood loss lower) whilst regular laboratory testing and improved clinical awareness are such that post-operative hyponatraemia is more likely to be identified than previously. At the same time, we have two potentially conflicting trends in peri-operative medical care. Firstly, older and comorbid patients are presenting for elective orthopaedic surgery. Secondly, we are moving towards shorter inpatient stays and even day case arthroplasty. This means we need to better identify those with underlying vulnerabilities who are at risk of hyponatraemia and other negative outcomes, whilst minimising unnecessary tests for those likely to tolerate a small and short-lived drop in Na concentration. Regular electrolyte testing reduces the risk of missing hyponatraemia; however, Kildow et al. (2018), have suggested that many such tests may be unnecessary (Kildow et al., 2018). Whilst thorough guidelines exist regarding identification and management of hyponatraemia which all reference, and are relevant to, post-operative hyponatraemia, specific guidance covering this area is lacking. The American guidelines report post-operative hyponatraemia as a syndrome of inappropriate antidiuretic hormone secretion (SIADH) and estimate a duration of 2–3 days (Verbalis et al., 2013).

There is currently a lack of evidence and guidance regarding the management of post-operative hyponatraemia for enhanced recovery programmes following arthroplasty. We have succeeded in reducing LOS whilst maintaining a high quality of evidence-based care. We aim to improve our service further, but this requires explicit understanding of the challenges we face. Implant survivorship is well reported in the United Kingdom (UK) (NJR, 2013; NJR, 2018). Medical complications have been investigated less often, a notable exception being the Scottish Arthroplasty Project, which records a small number of specific outcomes.

The aims of this audit were to establish (i) the incidence of and risk factors associated with hyponatraemia following elective primary total hip arthroplasty (THA), total knee arthroplasty (TKA), and uni-knee arthroplasty (UKA); (ii) the associations between post-operative hyponatraemia and other post-operative complications; and (iii) the natural history/timing of post-operative hyponatraemia in our population.

## Methods

Data were collected within a retrospective audit of inpatient complications and unplanned reattendance and readmission at hospital in consecutive elective primary hip and knee arthroplasty patients in a high throughput elective primary joint unit with the goal of improving patient care. The project was registered with the relevant Trust’s Standards, Quality and Audit Department. The hospital’s electronic database was used to identify 1000 patients who were admitted electively beginning 29th February 2012 (end date 25th June 2013) under the care of a single consultant orthopaedic surgeon for either THA, TKA, or UKA to a single surgical unit. At this time, spinal anaesthesia was given to most patients, and TKA and UKA patients routinely received a femoral nerve block. IV fluid was at the discretion of the anaesthetist. Routine discharge was on day 2 for THA and UKA patients and day 3 for TKA patients. A total of 1000 patients was arbitrarily chosen in an attempt to attain a representative sample size.

### Standard Surgical Technique and Peri-operative Practice

All THAs were performed in the lateral decubitus position using the posterior approach with a cementless Corail-Pinnacle combination (DePuy Synthes, Leeds UK). All TKAs were performed using a cementless low contact stress (LCS) rotating platform prosthesis without patellar resurfacing (DePuy Synthes, Leeds, UK). All UKA procedures were performed using the medial cementless Oxford uni-compartmental implant (Biomet, Warsaw, IN). Venous thromboembolism and antimicrobial prophylaxis were administered according to local policy at the time (Ogonda et al., 2016). Of note, antibiotic prophylaxis consisted of 2 g of flucloxacillin and 1.5 mg/kg of gentamicin given intravenously prior to knife to skin, followed by three further intravenous doses of 2 g of flucloxacillin at 6, 12, and 18 h post-operatively. Patients with a history of penicillin allergy or methicillin-resistant *Staphylococcus aureus* (MRSA) received 10 mg/kg teicoplanin and 1.5 mg/kg gentamicin intravenously once only prior to knife to skin. Tranexamic acid was administered to patients deemed at high risk of bleeding, including those with rapidly progressive osteoarthritis.

Routine practice during these time periods included routine checking of electrolytes on day 1 and day 2. Post-operative hyponatraemia was investigated and managed by the attending medical team. In Northern Ireland, medical staff are advised to manage hyponatraemia along standard lines using published guidelines (GAIN, 2010).

### Data Collection

Data were collected manually from individual clinical (medical and nursing) notes and local and regional electronic databases by trained staff, including nursing and medical staff. Data collected included baseline demographics, surgical and anaesthetic details, laboratory data, inpatient radiological imaging, inpatient post-operative complications (defined as an event requiring intervention by healthcare staff), LOS (based on date of admission and date of discharge), reattendance at emergency departments (ED), and/or readmission within 90 days and 90-day mortality.

Blood results were taken from the trust’s electronic laboratory system. This included surgery blood loss and blood loss at 24 and 48 hours post-operation. Pre-operative Na on the day of surgery was not available in all patients. When available, pre-operative Na, which was measured on the day of surgery, was taken. If this was not available, a pre-operative assessment Na was taken. Pre-operative assessment varied between 1 and 552 days before surgery, median 97 (interquartile range, IQR 65.3–141.0). Hyponatraemia was defined as a Na < 135 mmol/L. This was further categorised as mild, Na 130–134 mmol/L; moderate, Na 125–129 mmol/L; or severe, Na < 125 mmol/L (Spasovski et al., 2014). Of note, fasting glucose was checked for in a subset of patients only. This tended to be at physician request, but it is likely diabetics were overrepresented in the patients who had a fasting glucose checked.

### Statistical Analysis

All statistical analyses were carried out using SPSS for Windows (IBM, Version 22.0, Armonk, USA) and all data were assessed for normality using the Shapiro-Wilk test. Descriptive statistics, including absolute number (%) and median (IQR), were used as necessary. Chi-square analysis (or Fisher’s Exact test as appropriate) and Mann-Whitney U test were used to compare categorical and continuous variables, respectively, between those with and without post-operative hyponatraemia. Kruskal-Wallis testing was used to compare Na between THA, TKA, and UKA patients, since previous studies have found varying susceptibility to hyponatraemia by type of arthroplasty (Hennrikus et al. 2015, Sah, 2014). The Wilcoxin signed-rank test was used to compare pre- and post-operative Na. Logistic regression analysis was used to determine factors associated with hyponatraemia. Variables which displayed a p < 0.1 in univariate analysis were added to the multivariate logistic regression model. Otherwise, statistical significance was set at the p < 0.05 level.

## Results

A total of 1015 patients underwent elective primary hip or knee arthroplasty during the audit period. Of these, one underwent non-arthroplasty surgery, one was a duplicate record, and notes were not available for 13 patients, leaving 1000 patients. Patients undergoing more than one surgery during the audit period were considered as different audit entries. Thirty-one patients had two primary arthroplasties during the audit period. No patient had more than two surgeries. Of the 1000 joint replacements, 493 (49.3%) were primary THA, 425 (42.5%) were primary TKA and 82 (8.2%) were primary UKA. One of the most common post-operative complications identified was hyponatraemia.

### Incidence and predictors of post-operative hyponatraemia

Out of the total 1000 patients, 217 (21.7%) had post-operative hyponatraemia, i.e. Na < 135 mmol/L. Of these, 177 (81.6%) had mild (Na 130–134 mmol/L), 37 (17.1%) had moderate (Na 125–129 mmol/L), and 3 (1.4%) had severe (Na < 125 mmol/L) hyponatraemia. Three patients had post-operative hypernatraemia (Na > 145 mmol/L). For the purposes of the analyses, these patients were included in the non-hyponatraemia group.

Baseline characteristics and peri-operative variables for all 1000 patients are shown in Table 1 with a comparison of those with and without post-operative hyponatraemia. Of the 42 (4.2%) patients who had hyponatraemia pre-operatively (5 moderate and 37 mild), 31 (73.8%) also had post-operative hyponatraemia, of whom 3 had moderate pre-operative hyponatraemia which remained moderate, 27 had mild pre-operative hyponatraemia which remained mild, and one had mild pre-operative hyponatraemia which became severe. The remaining 11 (26.2%) who presented with hyponatraemia before surgery had a post-operative Na of ≥ 135 mmol/L (2 moderate and 9 mild, pre-operatively). The full list of medical complications by incidence in the hyponatraemia group and non-hyponatraemia group is provided in Table 2. Medication use for all 1000 patients and a comparison of those with and without post-operative hyponatraemia are shown in Table 3.

**Table 1:** Pre-operative patient characteristics for total group and comparison of those with and without post-operative hyponatraemia

	Hyponatraemia (Na < 135 mmol/L) post-operation (***n =*** 217)	No hyponatraemia (Na ≥ 135 mmol/L) post-operation (***n =*** 783)	***p***-value^**1**^	Total group (***n*** = 1000)
**Gender (*n =*** **1000)**				
Females	135 (62.2%)	453 (57.9%)	0.248	588 (58.8%)
Males	82 (37.8%)	330 (42.1%)		412 (41.2%)
**Age (years) (*n =*** **1000)**	73.0 (67.0–78.0)	68.0 (61.0–75.0)	**< 0.001**	69.0 (62.0–76.0)
**Height (cm) (*n*** **= 754)**	162.2 (155.7–169.5)	165.0 (158.5–173.0)	**0.001**	164.4 (158.0–172.5)
**Weight (kg) (*n*** **= 996)**	80.0 (67.6–92.0)	83.0 (72.0–95.4)	**0.035**	82.6 (71.0–94.1)
**BMI (kg/m**^**2**^**) (*n*** **= 991)**	29.8 (26.3–34.0)	29.9 (26.7–33.7)	0.657	29.9 (26.6–22.8)
**ASA grade (*n*** **= 1000)**				
1	7 (3.2%)	54 (6.9%)	**0.013**	61 (6.1%)
2	161 (74.2%)	595 (76.0%)		756 (75.6%)
3/4	49 (22.6%)	134 (17.5%)		183 (18.3%)
**Type of arthroplasty (*n*** **= 1000)**				
Hip	100 (46.1%)	393 (50.2%)	0.391	493 (49.3%)
Knee	101 (46.5%)	324 (41.4%)		425 (42.5%)
Uni-knee	16 (7.4%)	66 (8.4%)		82 (8.2%)
**Length of stay (*n*** **= 1000)**	5.0 (3.0–7.0)	3.0 (2.0–4.0)	**< 0.001**	3.0 (2.0–5.0)
**Pre-operative Na (mmol/L) (*n*** **= 999)**	138.0 (136.0–140.0)	140.0 (138.0–141.0)	**< 0.001**	139.0 (138.0–141.0)
**Blood loss during surgery (ml) (*n*** **= 481)**^**+**^	150.0 (100.0–212.5)	150.0 (100.0–250.0)	0.614	150.0 (100.0–243.0)
**24-h blood loss (ml) (*n*** **= 989)**	1133.7 (869.1–1515.2)	1109.0 (798.5–1402.4)	0.128	1113.7 (810.8–1428.1)
**48-h blood loss (ml) (*n*** **= 989)**	1390.3 (989.0–1817.9)	1264.8 (885.0–1633.9)	**0.003**	1287.7 (899.9–1675.8)
**Duration of operation (min) (*n*** **= 1000)**	53 (46–64)	55 (47–63)	0.265	54 (47–63)
**General anaesthetic (*n*** **= 1000)**	18 (8.3%)	68 (8.7%)	0.856	86 (8.6%)
**Spinal block (*n*** **= 1000)**	212 (97.7%)	769 (98.2%)	0.580	981 (98.1%)
**Peripheral nerve block (*n*** **= 1000)**	142 (65.4%)	464 (59.3%)	0.099	606 (60.6%)
**Transfusion post-operation (*n*** **= 961)**	44 (20.3%)	50 (6.4%)	**< 0.001**	94 (9.4%)
**Total intravenous fluids on day of surgery (ml) (*n*** **= 392)**	2500 (2210–3000)	2500 (2110–3000)	0.565	2500 (2140–3000)
**Pre-operative haemoglobin (g/L) (*n*** **= 894)**	131.0 (121.0–141.0)	136 (125.0–145.0)	**0.001**	134.0 (124.0–144.0)
**Fasting glucose (mmol/L) (*n*** **= 422)**	7.4 (6.4–8.6)	6.5 (5.9–7.6)	**< 0.001**	6.7 (5.9–7.8)

Median (IQR) used for continuous variables and n (%) used for categorical variables

Chi-square analysis used to compare categorical variables and Mann-Whitney U test used to compare continuous variables

^1^*p*-value comparing those with post-operative hyponatraemia (Na < 135 mmol/L) and those with normal sodium values (Na ≥ 135 mmol/L) post-operatively

^+^Blood loss during surgery was only available in hip replacement patients

For pre-operative Na, when available, Na on the day of surgery was taken, if this was not available, pre-assessment Na was taken

**Table 2:** Number of patients experiencing inpatient events following elective primary hip and knee arthroplasty

Post-operative inpatient events	Incidence total cohort (***n*** = 996)	Incidence hyponatraemia group (Na < 135 mmol/L) post-operation (***n*** = 217)	Incidence No hyponatraemia group (Na ≥ 135 mmol/L) post-operation (***n*** = 779)	***p***-value
**Cardiac**				
Chest pain	21 (2.1%)	*	17 (2.2%)	1.000^+^
Acute coronary syndrome	*	*	0 (0.0%)	**0.047**^+^
Arrhythmia	25 (2.5%)	16 (7.4%)	9 (1.2%)	**< 0.001**
Pulmonary oedema	24 (2.4%)	11 (5.1%)	13 (1.7%)	**0.004**
Cardiorespiratory arrest	*	0 (0.0%)	*	1.000^+^
**Respiratory**				
Exacerbation of airways disease	17 (1.7%)	*	15 (1.9%)	0.551^+^
Atelectasis	47 (4.7%)	12 (5.5%)	35 (4.5%)	0.524
Lower respiratory tract infection	49 (4.9%)	21 (9.7%)	28 (3.6%)	**< 0.001**
Pulmonary embolus	11 (1.1%)	8 (3.7%)	*	**< 0.001**^+^
Requirement for mechanical ventilation (invasive or non-invasive)	*	0 (0.0%)	*	1.000^+^
**Gastrointestinal (GI)**				
Antiemetic given^a^	351 (35.2%)	81 (37.3%)	270 (34.7%)	0.467
Constipation^b^	25 (2.5%)	*	41 (5.3%)	0.478
GI bleed	*	*	0 (0.0%)	0.218^+^
**Neurologica**l				
Confusion^c^	65 (6.5%)	22 (10.1%)	43 (5.5%)	**0.015**
Vasovagal episode^d^	249 (25.0%)	54 (24.9%)	195 (25.0%)	0.965
Fall	15 (1.5%)	5 (2.3%)	10 (1.3%)	0.340^+^
Cerebrovascular accident/transient ischaemic attack	*	0 (0.0%)	*	1.000^+^
**Miscellaneous**				
Requiring intravenous fluids	105 (10.5%)	23 (10.6%)	82 (10.5%)	0.975
Hyponatraemia — drop in Na < 5	157 (15.7%)	111 (51.2%)	46 (5.9%)	**< 0.001**
Acute kidney injury – Stage 1,2 or 3 KDIGO criteria	73 (7.3%)	31 (14.3%)	42 (5.4%)	**< 0.001**
Urinary tract infection	36 (3.6%)	10 (4.6%)	26 (3.3%)	0.375
Transfusion of blood products	94 (9.4%)	45 (20.7%)	49 (6.3%)	**< 0.001**
Difficult or delayed mobilisation^e^	134 (13.5%)	38 (17.5%)	96 (12.3%)	**0.048**
Wound-related complications^f^	99 (9.9%)	27 (12.4%)	72 (9.2%)	0.164
Delayed discharge awaiting social services	87 (8.7%)	25 (11.5%)	62 (8.0%)	0.100
Transferred to another unit for acute post-operative care	11 (1.1%)	5 (2.3%)	6 (0.8%)	0.068^+^
Transferred to another unit for rehabilitation	10 (1.0%)	*	8 (1.0%)	1.000^+^

All p-values provided by chi-square test apart from ^+^ which indicates Fisher’s Exact Test

*< 5 patients — number not stated to protect patient confidentiality

^a^Antiemetics may have been given prophylactically as well as to treat symptoms

^b^Laxative prescription was standard practice during this period. Constipation figures reflect instances where constipation required additional input during the inpatient journey

^c^Confusion includes delirium or confusion related to pre-existing cognitive impairments

^d^Vasovagal includes any instance where light-headedness on standing or mobilising necessitated limitations to mobilisation and/or return to bed. It also includes the less common episode of loss of consciousness

^e^Difficulty or delayed mobilisation includes people who were slow to mobilise for any reason including pre-existing deficits, knee braces, and/or resolving regional anaesthesia

^f^We used a very broad definition for wound-related complications include bleeding requiring change of dressing

**Table 3:** Medication use for total group and comparison of those with and without post-operative hyponatraemia

Medication	Hyponatraemia (Na < 135 mmol/L) post-operation (***n*** = 217)	No hyponatraemia (Na ≥ 135 mmol/L) post-operation (***n*** = 783)	***p***-value^**1**^	Total group (***n*** = 1000)
**Warfarin**	13 (6.0%)	26 (3.3%)	0.072	39 (3.9%)
**Other oral anticoagulants**	0 (0%)	7 (0.9%)	0.357	7 (0.7%)
**Antiplatelets**	79 (36.4%)	221 (28.2%)	**0.018**	300 (30%)
**Steroids**	7 (3.2%)	29 (3.7%)	0.747	36 (3.6%)
**Hypoglycaemic agents**	29 (13.4%)	54 (6.9%)	**0.002**	83 (8.3%)
**Insulin**	7 (3.2%)	9 (1.1%)	0.058	16 (1.6%)
**PPI/H**_**2**_ **receptor antagonist**	105 (48.4%)	344 (43.9%)	0.221	449 (44.9%)
**Bisphosphonate**	17 (7.8%)	68 (8.8%)	0.704	85 (8.5%)
**Oral calcium**	26 (12.0%)	69 (8.8%)	0.153	95 (9.5%)
**Levothyroxine**	36 (16.6%)	81 (10.3%)	**0.011**	117 (11.7%)
**Oral opioid analgesics**	66 (30.4%)	285 (36.3%)	0.111	351 (35.1%)
**Opioid analgesic patch**	18 (8.3%)	93 (11.9%)	0.142	111 (11.1%)
**NSAIDs**	68 (31.3%)	248 (31.7%)	0.957	316 (31.6%)
**ACEi/ARB**	107 (49.3%)	258 (33.3%)	**< 0.001**	365 (36.5%)
**Bendrofluazide/indapamide/CCB/alpha blocker**	99 (45.6%)	251 (32.1%)	**< 0.001**	350 (35.0%)
**B-blocker**	58 (26.7%)	155 (19.8%)	**0.025**	213 (21.3%)
**Antianginals, digoxin and loop and potassium sparing diuretics**	41 (18.9%)	105 (13.4%)	**0.040**	146 (14.6%)
**Statins**	97 (44.7%)	294 (37.5%)	0.130	391 (39.1%)
**Prostate drugs/drugs for incontinence**	16 (7.4%)	88 (11.2%)	0.103	103 (10.3%)
**Hypnotics/antidepressants/neuropathic pain/antiepileptics**	60 (27.6%)	259 (33.1%)	0.139	319 (31.9%)
**Dementia/PD drugs**	7 (3.2%)	16 (2.0%)	0.307	23 (2.3%)
**Inhalers**	28 (12.9%)	103 (13.2%)	0.923	131 (13.1%)
**Antibiotic type**				
**Flucloxacillin**	134 (61.8%)	565 (72.2%)	**0.003**	699 (69.9%)
**Gentamicin**	137 (63.1%)	568 (72.5%)	**0.007**	705 (70.5%)
**Teicoplanin**	80 (36.9%)	215 (27.5%)	**0.007**	295 (29.5%)

^1^*p*-value comparing those with post-operative hyponatraemia (Na < 135 mmol/L) and those with normal sodium values (Na ≥ 135 mmol/L) post-operatively

After adjusting for the factors which showed a significance of p < 0.1 in the univariate analysis, age, pre-operative Na and fasting glucose on day 1 remained significantly associated with having hyponatraemia post-operation (Table 4). Pre-operative Na (mmol/L) was the most statistically significant predictor. Both pre-operative Na and fasting glucose were treated as continuous variables. We found a reduced risk of post-operative hyponatraemia (OR = 0.66; 95% CI = 0.55–0.78) for every 1 mmol/L increase in pre-operative Na and an increased risk of post-operative hyponatraemia (OR = 1.33; 95% CI = 1.08–1.64) for every 1 mmol/L increase in fasting glucose on day 1 (Table 4).

**Table 4:** Logistic regression analysis of factors associated with having post-operative hyponatraemia following elective primary hip and knee arthroplasty

	Unadjusted Odds ratio (95% CI)	***p***-value	Adjusted Odds ratio (95% CI)*	***p***-value
**Age (years)**	1.05 (1.03–1.07)	**< 0.001**	1.07 (1.02–1.13)	**0.007**
**Height (cm)**	0.97 (0.95–0.99)	**0.001**	0.95 (0.93–1.01)	0.220
**Weight (kg)**	0.99 (0.98–1.00)	**0.019**	1.02 (0.99–1.05)	0.151
**ASA grade**				
**1 (reference)**	1.0 (reference)	-	1.0 (reference)	-
**2**	2.09 (0.93–4.68)	0.074	0.64 (0.24–1.78)	0.371
**3**	2.82 (1.20–6.62)	**0.017**	0.51 (0.13–1.66)	0.245
**Peripheral nerve block**	1.30 (0.95–1.78)	0.100	1.31 (0.56–3.02)	0.534
**Pre-operative Na (mmol/L)**	0.72 (0.67–0.77)	**< 0.001**	0.66 (0.55, 0.78)	**< 0.001**
**Fasting glucose (mmol/L)**	1.38 (1.21–1.56)	**< 0.001**	1.33 (1.08, 1.64)	**0.007**
**Pre-operative haemoglobin (g/L)**	0.98 (0.97–0.99)	**< 0.001**	0.98 (0.95, 1.01)	0.138
**Antibiotic**				
**Flucloxacillin**	0.62 (0.45, 0.85)	**0.003**	0.50 (0.21, 1.18)	0.112
**Gentamicin**^**+**^	0.65 (0.47, 0.89)	**0.007**		
**Teicoplanin**^**+**^	1.54 (1.12–2.12)	**0.007**		
**Medication**				
**Warfarin**	1.86 (0.94–3.68)	0.076	0.55 (0.08–3.69)	0.540
**Antiplatelets**	1.47 (1.07–2.02)	**0.018**	0.65 (0.24–1.71)	0.379
**Hypoglycaemic agents**	2.09 (1.30, 3.38)	**0.002**	1.64 (0.52, 5.16)	0.396
**Insulin**	2.88 (1.06–7.83)	**0.038**	0.81 (0.12–5.64)	0.835
**Levothyroxine**	1.73 (1.13–2.65)	**0.011**	1.15 (0.42–3.17)	0.790
**ACEi/ARB**	2.00 (1.47, 2.71)	**< 0.001**	1.31 (0.60, 2.86)	0.503
**Bendrofluazide/indapamide/**	1.79 (1.32, 2.44)	**< 0.001**	0.89 (0.41, 1.94)	0.769
**CCB/alpha blocker**				
**B-blocker**	1.49 (1.05–2.11)	**0.026**	2.54 (1.00–6.46)	0.050
**Antianginals, digoxin and loop and potassium sparing diuretics**	1.51 (1.02–2.25)	**0.041**	0.81 (0.31–2.14)	0.676

Logistic regression analysis used to determine factors associated with post-operative hyponatraemia (Na < 135 mmol/L)

*Adjusted for variables which showed a p-value < 0.10 in Tables 1 and 2; age, height, weight, ASA grade, peripheral nerve block, pre-operative Na, fasting glucose, pre-operative haemoglobin, flucloxacillin, warfarin, antiplatelets, hypoglycaemic agents, insulin, levothyroxine, ACEi/ARB, bendrofluazide/indapamide/CCB/alpha blocker, B-blocker and antianginals, digoxin and loop and potassium sparing diuretics

^+^Gentamicin and teicoplanin not included in multivariate analysis due to multicollinearity. Flucloxacillin kept in multivariate analysis using stepwise backwards approach

### Associations between post-operative hyponatraemia and other post-operative complications

Post-operative hyponatraemia was associated with several other post-operative complications in our population. Cardiac arrhythmia, pulmonary oedema, lower respiratory tract infection, pulmonary embolism, confusion, acute kidney injury, and blood transfusion were all more common in those with post-operative hyponatraemia (Table 2). These post-operative complications were also more common in those with post-operative mild hyponatraemia compared to those without post-operative hyponatraemia (results not shown). Further consideration was given to those clinical outcomes associated with hyponatraemia in the literature. Post hoc analysis was completed to determine whether hyponatraemia could be associated with confusion and falls. The number of patients experiencing confusion post-operatively was significantly higher (*p* = 0.015) in those with hyponatraemia (22/217 = 10.1%) compared to those without post-operative hyponatraemia (43/779 = 5.5%). This association did not withstand multivariate analysis (Supplementary Table 1). There was no significant difference (*p* = 0.340) in falls between those with hyponatraemia (5/217 = 2.3%) compared to those without post-operative hyponatraemia (10/779 = 1.28%). There was also no significant difference (*p* = 0.798) in vasovagal episodes between those with hyponatraemia (50/217 = 23.0%) compared to those without post-operative hyponatraemia (186/779 = 23.9%). Vasovagal episodes included any instance where light-headedness on standing or mobilising necessitated limitations to mobilisation and/or return to bed, as well as the less common episodes of loss of consciousness.

A significantly higher proportion (p < 0.001) of those with hyponatraemia (138/217 = 63.6%) had a delayed discharge (LOS over 3 days) compared to those without hyponatraemia (300/779 = 38.5%). However, in multivariate analysis, after adjustment for age, pre-operative Na, post-operative Na, ASA grade, type of surgery, transfusion, post-operative confusion, arrhythmia, pulmonary oedema, lower respiratory tract infection, pulmonary embolism, and acute kidney injury, post-operative Na was no longer associated with delayed discharge (Supplementary Table 2).

There were no significant differences (*p* = 0.072) in reattendance at ED, and/or readmission within 90 days between those who had post-operative hyponatraemia (39/217 = 18.0%) and those who did not (103/783 = 13.2%). There were also no significant differences (*p* = 0.471) in reattendance at ED, and/or readmission within 90 days between those who were discharged with hyponatraemia (18/108 = 16.7%) and those discharged with normal Na (124/880 = 14.1%). Reasons for reattendance at ED, and/or readmission within 90 days in those with and without post-operative hyponatraemia are provided in Supplementary Table 3. No patients died within 90 days.

### The natural history/timing of post-operative hyponatraemia in our population

Table 5 provides the Na concentrations for the total 1000 patients with a comparison by arthroplasty type and with and without post-operative hyponatraemia. Of the 108 patients discharged with Na < 135mmol/L, 99 patients had mild hyponatraemia, eight patients had moderate hyponatraemia, and one patient had severe hyponatraemia.

**Table 5:** Serum sodium concentrations for total group, arthroplasty type and with and without post-operative hyponatraemia

	Hip (***n*** = 493)	Knee (***n*** = 425)	Uni-knee (***n*** = 82)	***p***-value^**1**^	Hyponatraemia (Na < 135 mmol/L) post-operation (***n*** = 217)	No hyponatraemia (Na ≥ 135 mmol/L) post-operation (***n*** = 783)	***p***-value^**2**^	Total (***n*** = 1000)
**Post-operative Na < 135 mmol/L (*n*** **= 1000)**	100 (20.2%)	101 (23.8%)	16 (19.5%)	0.391	-	-	-	217 (21.7%)
**Post-operative Na decrease ≥ 5 mmol/L (*n*** **= 1000)**	*54 (11.0%)	*90 (21.2%)	13 (15.9%)	**< 0.001**	111 (51.1%)	106 (13.5%)	**< 0.001**	157 (15.7%)
**Pre-assessment Na (mmol/L) (*n*** **= 999)**	139.0 (138.0–141.0)	140.0 (138.0–141.0)	140.0 (138.0–141.0)	0.129	138.0 (136.0–140.0)	140.0 (138.0–141.0)	**< 0.001**	139.0 (138.0–141.0)
**Pre-operative Na (mmol/L) (*n*** **= 723)**	139.0 (138.0–141.0)	139.0 (138.0–141.0)	140.0 (138.0–141.0)	0.807	138.0 (136.0–140.0)	140.0 (138.0–141.0)	**< 0.001**	139.0 (138.0–141.0)
**Day 1 Na (mmol/L) (*n*** **= 994)**	138.0 (136.0–139.0)	138.0 (136.0–139.0)	138.0 (136.0–139.0)	0.794	134.0 (133.0–136.0)	138.0 (137.0–139.0)	**< 0.001**	138.0 (136.0–139.0)
**Day 2 Na (mmol/L) (*n*** **= 501)**	*137.0 (135.0–139.0)	*137.0 (134.0–139.0)	137.0 (135.0–140.0)	**0.039**	134.0 (132.0–135.0)	138.0 (137.0–140.0)	**< 0.001**	137.0 (135.0–139.0)
**Minimum post-operative Na (mmol/L) (*n*** **= 1000)**	137.0 (135.0–138.0)	137.0 (135.0–138.0)	137.0 (135.0–138.0)	0.475	133.0 (131.0–134.0)	138.0 (136.0–139.0)	**< 0.001**	137.0 (135–138.0)
**Na prior to discharge (mmol/L) (*n*** **= 988)**	138.0 (136.0–139.0)	137.0 (135.0–139.0)	138.0 (136.0–139.0)	0.332	134.0 (133.0–137.0)	138.0 (137.0–139.0)	**< 0.001**	138.0 (136.0–139.0)
**Change in Na (mmol/L) (*n*** **= 999)**	*– 2.0 (– 4.0 to – 1.0)	*– 3.0 (– 5.0 to – 1.0)	– 3.0 (– 4.0 to – 1.0)	**0.012**	– 6.0 (– 8.0 to – 4.0)	– 2.0 (– 3.0 to – 1.0)	**< 0.001**	– 3.0 (– 4.0 to – 1.0)
**Blood transfusion**	63 (12.4%)	31 (7.3%)	0 (0.0%)	**< 0.001**	44 (20.3%)	50 (6.4%)	**< 0.001**	94 (9.4%)
**24-h blood loss (*n*** **= 989)**	*1252.4 (972.6–1585.0)	*1064.5 (809.7–1320.8)	*535.2 (308.1–763.6)	**< 0.001**	1133.7 (869.1–1515.2)	1109.0 (798.5–1402.4)	0.128	1113.7 (810.8–1428.1)
**48-h blood loss (*n*** **= 989)**	*1405.2 (1017.8–1794.8)	*1264.7 (929.9–1567.1)	*646.2 (357.5–808.9)	**< 0.001**	1390.3 (989.0–1817.9)	1264.8 (885.0–1633.9)	**0.003**	1287.7 (899.9–1675.8)

Median (IQR) used for continuous variables and n (%) used for categorical variables

Kruskal-Wallis test used to compare continuous variables between arthroplasty type and Mann-Whitney U test used to compare continuous variables between those with and without post-operative hyponatraemia

*Groups which show significant difference

^1^*p*-value comparing those who underwent total hip arthroplasty, total knee arthroplasty and uni-compartmental knee arthroplasty

^2^*p*-value comparing those with post-operative hyponatraemia (Na < 135 mmol/L) and those with normal sodium values (Na ≥ 135 mmol/L) post-operatively

**Fig. 1:**
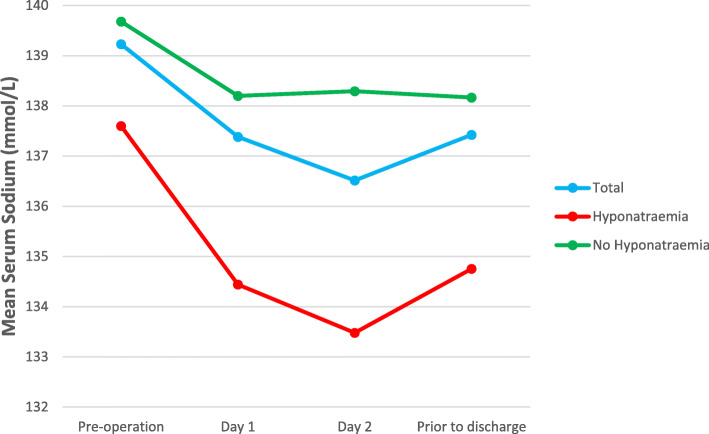
Mean serum sodium for total group and comparison of those with and without post-operative hyponatraemia

**Fig. 2:**
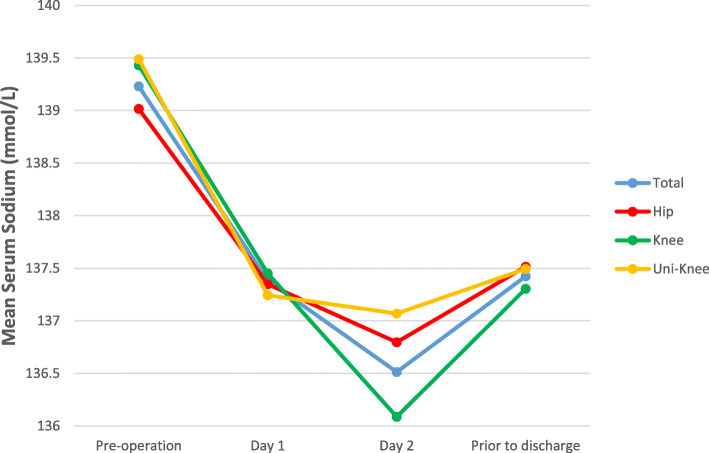
Mean serum sodium for total group and comparison of arthroplasty type

**Fig. 3:**
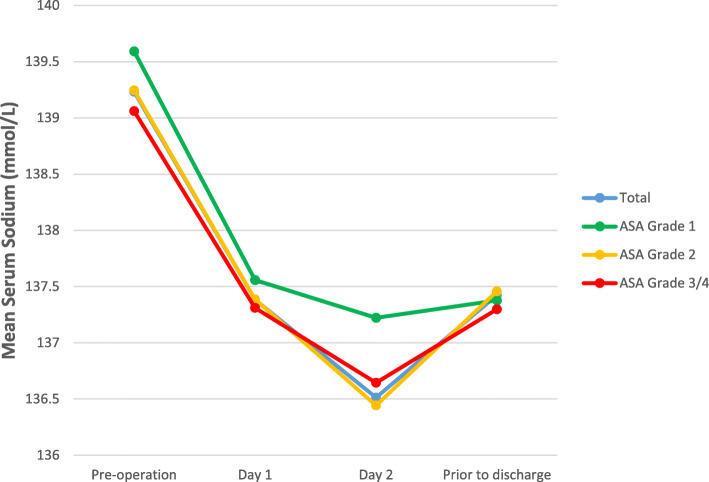
Mean serum sodium for total group and comparison of ASA grade

Figures 1, 2, and 3 show the mean Na at pre-operation, day 1, day 2, and last Na prior to discharge (Fig. 1, total group and comparison of those with and without post-operative hyponatraemia; Fig. 2, total group and comparison of arthroplasty type; and Fig. 3, total group and comparison of ASA grade).

## Discussion

### Main findings

We have found (i) significant associations between age, pre-operative Na concentration, fasting glucose on day 1, and post-operative hyponatraemia; (ii) that post-operative hyponatraemia is associated with other post-operative complications; (iii) no difference between reattendance at ED and/or readmission rates in those who developed or were discharged with post-operative hyponatraemia; and (iv) people developing post-operative hyponatraemia started from a lower pre-operative Na, and had a bigger drop in Na, than those maintaining a normal Na concentration peri-operatively.

Sah (2014) found a higher incidence of hyponatraemia (40%) in their elective joint arthroplasty population. Similar to our findings, they also found risk factors of post-operative hyponatraemia included pre-operative hyponatremia and older age. They also found female sex, lower body weight, knee in comparison to hip surgery, and bilateral knee arthroplasty to be risk factors. Our finding that age and pre-operative Na concentration independently predicts post-operative hyponatraemia supports the understanding of post-operative hyponatraemia, in this population, as a result of both baseline physiological vulnerability and peri-operative changes. Leung et al. (2012), in their database study, showed that pre-operative hyponatraemia (Na < 135 mmol/L) predicted longer LOS and increased risk of post-operative complications and 30-day mortality (Leung et al., 2012). Abola et al. (2019) also found that pre-operative hyponatraemia was associated with longer LOS and greater risk of reoperation (Abola et al., 2019). In our study, we found a significantly longer length of stay in those with post-operative hyponatraemia; however, pre- or post-operative sodium levels were not associated with a delayed discharge in multivariate analysis. We also found no significant differences in reattendance at ED, and/or readmission within 90 days between those who had post-operative hyponatraemia and those who did not.

That hyperglycaemia can lower Na is well-established. Traditional formulae for correcting Na for hyperglycaemia (Katz, 1973; Hillier et al., 1999) predict a smaller change in sodium than what was observed here, suggesting that there may be other contributory mechanisms. Diabetes mellitus has been associated with an increased incidence of hyponatraemia in community studies (Liamis et al., 2013; Mohan et al., 2013) and with increased ADH levels. Hyperglycaemia following arthroplasty has, to date, largely been studied for its association with joint infection (Hwang et al., 2015).

Intra-operative antibiotic use of teicoplanin was associated with a higher incidence of hyponatraemia compared to flucloxacillin and gentamicin. Due to multicollinearity, and using a stepwise backwards approach, flucloxacillin was no longer significantly associated with post-operative hyponatraemia in the multivariate analysis, whilst gentamicin and teicoplanin were excluded from the adjusted model.

Those with post-operative hyponatraemia did have higher rates of inpatient complications which may have contributed to their longer LOS. It must be acknowledged that several of the complications studied (e.g. pulmonary oedema and acute kidney injury) may be reflective of fluid balance issues and are therefore not mutually exclusive to hyponatraemia. Cardiovascular medications were significantly associated with hyponatraemia in univariate analysis, although they lost their significance in the multivariate analysis. Previous findings have reported that cardiovascular medications predict post-operative hyponatraemia (Udy et al., 2005; Hawkins et al., 2003; Mohan et al., 2013; Bissram et al., 2007). The relevant drugs are indicated for both hypertension and heart failure. It is not clear whether these associations between drugs and post-operative hyponatraemia are due to the drugs themselves, or are by virtue of underlying comorbidities predisposing to hyponatraemia. A propensity for heart failure and fluid retention could exacerbate the appropriate peri-operative release of ADH that is likely the main driver of post-operative hyponatraemia. Of note, our practice changed during the time of this audit and instead of being judged on a case-by-case basis, a blanket withholding of angiotensin converting enzyme inhibitors and angiotensin receptor blockers, in particular over the peri-operative period, was introduced. We found decreased height and decreased weight in those with post-operative hyponatraemia, but no significant difference in BMI between those with post-operative hyponatraemia and those with normal Na. Sah (2014) also reported lower body weight in those with post-operative hyponatraemia. It is possible that those with lower height and weight were subject to a relative excess of intravenous fluids infusion post-operatively. Whilst we have included infused fluid volumes (on day of surgery, available for *n* = 392 only) in our analyses (Table 1), we did not have net fluid balance available for sufficient patients to do this.

It is noteworthy that there was no significant increase in reattendance at ED and/or readmission to hospital for those patients who developed post-operative hyponatraemia. Our rates of discharge with hyponatraemia (10.8%) were lower than those reported in other series, with Hennrikus et al. (2015) reporting 28% of patients to still be hyponatraemic on discharge from hospital (Hennrikus et al., 2015). Hennrikus et al. (2015) included not only THA and TKA but also spine fusion and other surgeries, such as shoulder arthroplasty and foot and ankle procedures. Our findings, therefore, challenge the rationale for keeping otherwise well patients in hospital solely to monitor Na. It has been suggested that the standard reference range for Na utilised by most laboratories may not be universally applicable to inpatients (McKee et al., 2016). Our data would support this concept and the notion that milder degrees of hyponatraemia are common and probably do not require extensive investigation or treatment. We suggest that otherwise well patients with mild hyponatraemia can safely be discharged and followed up in the community. It is important to note that this is an observational finding. Evidence directly comparing inpatient and outpatient management of post-operative hyponatraemia is required.

The association between post-operative hyponatraemia and confusion did not withstand multivariate analysis, suggesting other factors such as age, pre-operative Na, ASA grade, type of surgery, and transfusion may have been mediating the relationship. It should be noted that we were not able to further categorise confusion, which may have been due to delirium, or pre-existing cognitive impairment. It is also likely, given other estimates from our population (Cunningham et al., 2017), that the incidence of confusion, due to either cause, was underestimated in this audit. There were no significant differences in falls or vasovagal episodes between those with and those without hyponatraemia. This is in contrast to the findings of a systematic review by Corona et al. (2018), which reported hyponatraemia to be significantly associated with an increased risk of falls across all 15 included studies (Corona et al., 2018). Ahamed et al. (2014) also found hyponatraemia to be independently associated with increased risk of admission-associated falls in their case-control study of medical inpatients (Ahamed et al., 2014).

The lack of effect of duration of surgery or blood loss during surgery on post-operative hyponatraemia in our cohort is contrary to other reports (Hennrikus et al., 2015). Our values of both are low (150 ml of blood loss in those with and those without hyponatraemia, and 53 min duration of surgery for those with hyponatraemia vs 55 min for those without hyponatraemia), suggesting we may be below the threshold where it makes a difference. For example, Hennrikus et al. (2015) noted significant differences in both duration of surgery and blood loss; duration of surgery was 2.6 h in those with no hyponatraemia vs 2.9 h in those who developed hyponatraemia post-operatively, and blood loss was 261 ml in those with no hyponatraemia vs 332 ml in those who developed hyponatraemia post-operatively (Hennrikus et al., 2015). Gender differences have previously been described in some studies, with females at a higher risk of hyponatraemia than males (Mohan et al., 2013), whilst other studies have found no gender differences (Hawkins et al., 2003), as in this audit.

### Strengths

Strengths of this study include the high numbers of participants with clinical chart-level information, including pre-operative drug information. The patients included in this audit are representative of the UK arthroplasty population with a similar mean age, gender ratio, and ASA grading to the National Joint Registry (NJR) population captured in the 10th Annual NJR Report 2013 for England, Wales and Northern Ireland (NJR, 2013) (Supplementary Table 4). A wealth of patient level data not included in other studies, or captured by the electronic systems on which larger studies are based, are presented here.

### Limitations

Available pre-operative Na measurements were taken in the 24 h prior to surgery for 723 patients, but for 276 patients, pre-operative Na was taken from the pre-operative assessment which varied between 1 and 552 days before surgery. The last recorded Na prior to discharge may have been taken more than 24 h prior to discharge. Variables differed in the extent of missing data, with some variables having more missing data than others.

Serum and urinary osmolarities, treatment of post-operative hyponatraemia, for example, fluid restriction, administered intravenous fluid volumes and daily fluid balance, and whether cardiac drugs were held over the peri-operative period have not been described. A validated comorbidity score has not been used pre-operatively, although ASA grade and medications can give some indication of pre-operative comorbidity. Information regarding complications was recorded pragmatically from the notes and strict criteria were not used. This was a single-surgeon, single-centre series which could influence generalisability.

## Conclusion

In this audit of 1000 THA, TKA, and UKA patients, 217 (21.7%) had post-operative hyponatraemia. Post-operative hyponatraemia can, to an extent, be predicted. We found no evidence that those discharged with hyponatraemia have more reattendance at ED or readmission to hospital. As we move towards better planned and more efficient inpatient stays for arthroplasty patients, comprehensive pre-operative risk assessment is necessary and must take age, pre-operative Na, and medications into account. Post-operative monitoring and management of post-operative hyponatraemia must be tailored to the individual. We propose that mild (Na 130–134 mmol/L) hyponatraemia may be of little to no consequence in the immediate post-operative period and suggest that further investigation would be valuable to determine if moderate (Na 125–129 mmol/L) hyponatraemia in otherwise well individuals can be managed in the community. Evidence directly comparing management strategies would be preferable.

## Supplementary Information

**Additional file 1: Supplementary Table 1.** Logistic regression analysis of factors associated with having confusion following elective primary hip and knee arthroplasty. **Supplementary Table 2.** Logistic regression analysis of factors associated with having a delayed discharge (length of stay over 3 days) following elective primary hip and knee arthroplasty. **Supplementary Table 3.** Reasons for reattendance/readmission in those with post-operative hyponatraemia. **Supplementary Table 4.** Comparison of demographics of hip and knee replacements patients in the current audit of 1,000 Joints and the 10th Annual National Joint Registry Report 2013

## Data Availability

The datasets used and/or analysed during the current study are available from the corresponding author on reasonable request.

## Abbreviations

ADH: Antidiuretic hormone
LOS: Length of stay
SIADH: Syndrome of inappropriate antidiuretic hormone secretion
UK: United Kingdom
THA: Total hip arthroplasty
TKA: Total knee arthroplasty
UKA: Uni-compartmental knee arthroplasty
LCS: Low contact stress
MRSA: Methicillin-resistant *Staphylococcus aureus*
GAIN: Guidelines and Audit Implementation Network
