# Supplementary material for: Prevalence, risk factors, and complications associated with hyponatraemia following elective primary hip and knee arthroplasty
Source: Perioper Med (Lond). 2021 Aug 3;10:25. doi: 10.1186/s13741-021-00197-1 (PMC8330106; doi:10.1186/s13741-021-00197-1)
Supplement: Supplementary file 1 — Additional file 1: Supplementary Table 1. Logistic regression analysis of factors associated with having confusion following elective primary hip and knee arthroplasty. Supplementary Table 2. Logistic regression analysis of factors associated with having a delayed discharge (length of stay over 3 days) following elective primary hip and knee arthroplasty. Supplementary Table 3. Reasons for reattendance/readmission in those with post-operative hyponatraemia. Supplementary Table 4. Comparison of demographics of hip and knee replacements patients in the current audit of 1,000 Joints and the 10th Annual National Joint Registry Report 2013 [file 13741_2021_197_MOESM1_ESM.docx]

**Supplementary Material**

**Supplementary Table 1.** Logistic regression analysis of factors associated with having confusion following elective primary hip and knee arthroplasty

|  | **Unadjusted Odds ratio (95% CI)** | **p-value** | ***Adjusted Odds ratio (95% CI)** | **p-value** |
| --- | --- | --- | --- | --- |
| **Age (years)** | 1.14 (1.10, 1.19) | **<0.001** | 1.13 (1.09, 1.17) | **<0.001** |
| **Pre-operative Na (mmol/L)** | 0.97 (0.88, 1.07) | 0.532 | 1.04 (0.93, 1.16) | 0.465 |
| **Post-operative Na (mmol/L)** | 0.92 (0.85, 0.98) | **0.016** | 0.98 (0.89, 1.08) | 0.676 |
| **ASA grade**  **1 (reference)**  **2**  **3/4** | 1.0 (reference)  1.74 (0.41, 7.38)  3.85 (0.88, 16.92) | **-**  0.451  0.074 | 1.0 (reference)  0.46 (0.10, 2.14)  0.61 (0.12, 3.02) | -  0.324  0.543 |
| **Type of Surgery**  **Hip (reference)**  **Knee**  **Uni-Knee** | 1.0 (reference)  1.61 (0.95, 2.73)  0.92 (0.31, 2.70) | -  0.074  0.875 | 1.0 (reference)  1.88 (1.05, 3.35)  1.41 (0.45, 4.47) | -  **0.033**  0.556 |
| **Transfusion** | 4.78 (2.67, 8.59) | **<0.001** | 2.43 (1.20, 4.92) | **0.014** |
| **Length of stay** | 1.13 (1.06, 1.21) | **<0.001** | 1.04 (0.96, 1.12) | 0.330 |

Logistic regression analysis used to determine factors associated with having confusion.

*Adjusted for age (years), pre-operative Na (mmol/L), post-operative Na (mmol/L), ASA grade, type of surgery, transfusion and length of stay.

**Supplementary Table 2.** Logistic regression analysis of factors associated with having a delayed discharge (length of stay over 3 days) following elective primary hip and knee arthroplasty

|  | **Unadjusted Odds ratio (95% CI)** | **p-value** | ***Adjusted Odds ratio (95% CI)** | **p-value** |
| --- | --- | --- | --- | --- |
| **Age (years)** | 1.08 (1.06, 1.09) | **<0.001** | 1.05 (1.04, 1.07) | **<0.001** |
| **Pre-operative Na (mmol/L)** | 0.93 (0.89, 0.98) | **0.004** | 1.00 (0.94, 1.07) | 0.919 |
| **Post-operative Na (mmol/L)** | 0.87 (0.83, 0.90) | **<0.001** | 0.94 (0.87, 1.02) | 0.131 |
| **ASA grade**  **1 (reference)**  **2**  **3/4** | 1.0 (reference)  3.07 (1.58, 6.00)  9.55 (4.64, 19.68) | **-**  **0.001**  **<0.001** | 1.0 (reference)  1.57 (0.77, 3.22)  3.77 (1.73, 8.23) | -  0.214  **0.001** |
| **Type of Surgery**  **Hip (reference)**  **Knee**  **Uni-Knee** | 1.0 (reference)  1.27 (0.97, 1.64)  0.71 (0.44, 1.16) | -  0.078  0.172 | 1.0 (reference)  1.29 (0.95, 1.76)  0.70 (0.40, 1.23) | -  0.099  0.217 |
| **Transfusion** | 7.95 (4.51, 14.03) | **<0.001** | 4.25 (2.24, 8.05) | **<0.001** |
| **Confusion** | 3.08 (1.79, 5.30) | **<0.001** | 1.44 (0.76, 2.74) | 0.265 |
| **Arrhythmia** | 9.78 (2.91, 32.91) | **<0.001** | 3.98 (1.05, 15.17) | **0.043** |
| **Pulmonary oedema** | 6.63 (2.25, 19.53) | **0.001** | 2.14 (0.64, 7.19) | 0.220 |
| **Lower respiratory tract infection** | 8.35 (3.71, 18.78) | **<0.001** | 6.73 (2.79, 16.20) | **<0.001** |
| **Pulmonary embolus** | 13.01 (1.66, 102.06) | **0.015** | 7.87(0.92, 67.42) | 0.060 |
| **Acute kidney injury – Stage 1,2 or 3 KDIGO criteria** | 2.97 (1.96, 4.50) | **<0.001** | 2.25 (1.39, 3.62) | **0.001** |

Logistic regression analysis used to determine factors associated with having a delayed discharge (length of stay over 3 days).

*Adjusted for age (years), pre-operative Na (mmol/L), post-operative Na (mmol/L), ASA grade, type of surgery, transfusion, confusion, arrhythmia, pulmonary oedema, lower respiratory tract infection, pulmonary embolus and acute kidney injury – Stage 1,2 or 3 KDIGO criteria.

**Supplementary Table 3.** Reasons for reattendance/readmission in those with post-operative hyponatraemia

| **Reason for reattendance/readmission** | **Number of patients with hyponatraemia** | **Number of patients without hyponatraemia** |
| --- | --- | --- |
| Deep Vein Thrombosis | 11 | 31 |
| Gastrointestinal upset | 4 | 11 |
| Elective surgery | 3 | 10 |
| Dislocation | 3 | 5 |
| Congestive heart failure | 2 | 0 |
| Fall/injury | 2 | 6 |
| Lower Respiratory Tract Infection | 2 | 5 |
| Pain | 2 | 2 |
| Chest pain | 1 | 3 |
| Shortness of Breath and chest pain | 1 | 1 |
| Haematuria | 1 | 0 |
| Hip pain | 1 | 2 |
| Hot swollen knee | 1 | 0 |
| Overdose | 1 | 0 |
| Pulmonary Embolism | 1 | 6 |
| Pyelonephritis | 1 | 0 |
| Wound infection | 1 | 1 |
| Urinary Tract Infection | 1 | 3 |
| Cellulitis | 0 | 1 |
| Palpitations and hallucinations | 0 | 1 |
| Leg swelling | 0 | 2 |
| Epistaxis | 0 | 1 |
| Atrial fibrillation | 0 | 1 |
| Transient ischemic attack | 0 | 1 |
| Nonspecific symptoms | 0 | 2 |
| Tremor | 0 | 1 |
| INR check | 0 | 1 |
| Flare arthritis | 0 | 1 |
| Finger infection | 0 | 1 |
| Request for crutches | 0 | 1 |
| Symptomatic anaemia | 0 | 1 |
| Small Bowel obstruction | 0 | 1 |
| Syncope | 0 | 1 |
| **Total** | **39** | **103** |

**Supplementary Table 4.** Comparison of demographics of hip and knee replacements patients in the current audit of 1,000 Joints and the 10th Annual National Joint Registry Report 2013

|  | **Current Audit of 1,000 Joints** | | **10th Annual National Joint Registry Report 2013** | |
| --- | --- | --- | --- | --- |
|  | **Hip** | **Knee** | **Hip** | **Knee** |
| **Age** | 68.5 (10.3) | 69.3 (9.4) | 68.7 (11.4) | 69.3 (9.7) |
| **Female** | 58% | 60% | 60% | 57% |
| **Male** | 42% | 40% | 40% | 43% |
| **ASA Grade**  **1**  **2**  **3**  **4 & 5** | 9%  73%  18%  0% | 4%  78%  18%  <1% | 15%  70%  15%  <1% | 11%  74%  15%  <1% |

Mean (SD) used for continuous variables and % used for categorical variables.
